# Clinical predictors of lung function in patients recovering from mild COVID-19

**DOI:** 10.1186/s12890-022-02086-9

**Published:** 2022-07-31

**Authors:** Arturo Cortes-Telles, Esperanza Figueroa-Hurtado, Diana Lizbeth Ortiz-Farias, Gerald Stanley Zavorsky

**Affiliations:** 1 Clínica de Enfermedades Respiratorias, Hospital Regional de Alta Especialidad de La Peninsula de Yucatan, Calle 7 #433 por 20 y 22. Fracc. Altabrisa, Mérida, Yucatan Mexico; 2 Department of Physiology and Membrane Biology, University of California, Davis, USA

**Keywords:** Respiratory function test, SARS-CoV-2, Mexico, DLCO, Lung

## Abstract

**Background:**

Few studies have assessed lung function in Hispanic subjects recovering from mild COVID-19. Therefore, we examined the prevalence of impaired pulmonary diffusing capacity for carbon monoxide (DLCO) as defined by values below the lower limit of normal (< LLN, < 5th percentile) or less than 80% of predicted in Hispanics recovering from mild COVID-19. We also examined the prevalence of a restrictive spirometric pattern as defined by the ratio of forced expiratory volume in 1 s (FEV_1_) to forced vital capacity (FVC) being ≥ LLN with the FVC being < LLN. Finally, we evaluated previous studies to find factors correlated to impaired DLCO post-COVID-19.

**Methods:**

In this observational study, adult patients (n = 146) with mild COVID-19 were recruited from a long-term follow-up COVID-19 clinic in Yucatan, Mexico, between March and August 2021. Spirometry, DLCO, and self-reported signs/symptoms were recorded 34 ± 4 days after diagnosis.

**Results:**

At post-evaluation, 20% and 30% of patients recovering from COVID-19 were classified as having a restrictive spirometric pattern and impaired DLCO, respectively; 13% had both. The most prevalent reported symptoms were fatigue (73%), a persistent cough (43%), shortness of breath (42%) and a blocked/runny nose (36%). Increased age and a restrictive spirometric pattern increased the probability of having an impaired DLCO while having a blocked nose and excessive sweating decreased the likelihood. The proportion of patients with previous mild COVID-19 and impaired DLCO increased by 13% when the definition of impaired DLCO was < 80% predicted instead of below the LLN. When comparing previous studies, having severe COVID-19 increased the proportion of those with impaired DLCO by 21% compared to those with mild COVID-19.

**Conclusions:**

One-third of patients with mild COVID-19 have impaired DLCO thirty-four days post-diagnosis. The criteria that define impaired DLCO and the severity of COVID-19 disease affects the proportion of those with impaired DLCO at follow-up. One-fifth of patients have a restrictive spirometric pattern.

**Supplementary Information:**

The online version contains supplementary material available at 10.1186/s12890-022-02086-9.

## Introduction

Severe acute respiratory syndrome coronavirus 2 (SARS-CoV2) is a virus that originated in Wuhan City, China, in December of 2019 and is responsible for the coronavirus disease (COVID-19) [1]. Between December 29, 2019, and February 16, 2020, deaths increased from 1 to 1666 in China.^1^ By October 1, 2021, over 4.79 million people worldwide have died from COVID-19 [2]. As such, COVID-19 has become one of the fatal pandemics ever recorded in human history.

As the number of patients recovering from COVID-19 increases worldwide, there is an urgent need to keep analysing pulmonary sequelae to facilitate optimal clinical treatments. COVID-19 is a heterogeneous disease with several long-term sequelae [3]. Patients recovering from the acute phase may report long-term multi-system symptoms, including various pulmonary function abnormalities [3–22], psychological sequelae [3], and reduced physical functioning [10, 23, 24]. Specifically, pulmonary diffusing capacity for carbon monoxide (DLCO) is significantly impaired 30 to 180 days after the onset of (SARS-CoV-2) (Additional file 1: Table S1). However, most of these studies evaluated lung function in hospitalised patients due to the severity of their condition; very few studies focused on patients recovering from mild COVID-19 [4, 7, 10, 20]. Furthermore, even fewer studies focus on the effects of COVID-19 in Latino populations [6, 25].

Recent reports indicate the presence of racial and ethnic disparities with a disproportionate burden of COVID-19-related severity infections and mortality [26, 27]. These disparities may be partly attributable to higher comorbidities that worsen COVID-19 outcomes [28]. Specifically, there is limited research on the physiological effects of COVID-19 in the Mexican Latino population. Few studies have analysed the Latino community's persistent symptoms and lung function post-COVID-19 [28].

Our main objectives were to (1) determine factors associated with an impaired DLCO in Hispanic patients with mild COVID-19; and (2) evaluate data from previous studies to determine which factors predicted the proportion of patients with an impaired DLCO at follow-up.

## Materials and methods

### Study design

This is an observational cross-sectional study from the Long-term follow-up COVID-19 clinic at the High Specialty Regional Hospital of Yucatan, Mexico, from March 2021 to August 2021. We consecutively enrolled one hundred and fifty patients during the period. Inclusion criteria were adults over 18 years old recovering from mild COVID-19, defined as symptomatic patients meeting the case definition for COVID-19 without evidence of pneumonia or hypoxia (current WHO diagnostic criteria) [29]. All patients were scheduled after 4–6 weeks (34.4 ± 3.8 days) of baseline symptoms to perform pulmonary function tests and were evaluated for persistent symptoms at the clinic. The Ethics Committee of the High Specialty Regional Hospital in Yucatan, Mexico, approved this study (Protocol number 2020–024). All patients provided written informed consent to participate in this study, in compliance with the Helsinki declaration. This study followed the Guidelines for Strengthening the Reporting of Observational Studies in Epidemiology (STROBE).

### Methods

Patients received a comprehensive medical assessment with a detailed medical history. Data including demographics, persistent symptoms from surveys, and pulmonary function test results were collected during the follow-up visit. Demographic data included age, sex, body mass index (BMI), previous cardiovascular disease risk factors for which regular pharmacological treatment was incorporated (including systemic hypertension, cardiac disease, diabetes mellitus), tobacco use (current or former smoker vs never smoker), obesity (defined as body mass index > 30 kg/m^2^). Patients were asked if they received oral corticosteroids and/or anticoagulants during the disease and to recount the presence or absence of symptoms at the time of the visit, including fatigue, shortness of breath on effort, cough, chest tightness, chest pain, sore throat, blocked and/or runny nose, loss of smell, loss of taste, diarrhoea, abdominal pain, muscle or joint pain, headache, tachycardia, sore or red eyes, excessive sweating (over a 24 h period, including night sweats), hair loss, and weight loss [30, 31].

Spirometry and measurements of DLCO were performed by a well-trained respiratory therapist who is also a Registered Pulmonary Function Technologist certified by The National Board for Respiratory Care. All tests were performed under physician supervision. The equipment used for lung function measurements was the Easy One Pro®, NDD Medical Technologies, Switzerland. Spirometry reference equations were obtained from Hankinson (1999) [32]. The technical quality of spirometry was adhered to per 2019 spirometry standards [33]. The reference equations for pulmonary diffusing capacity for carbon monoxide (DLCO) were obtained from Vazquez-Garcia and colleagues [32]. The 2017 Technical standards for DLCO were followed for technical quality [34].

### Analysis

Continuous variables are expressed ad mean (S.D.), and categorical variables are expressed as absolute values and percentages. A Fisher’s exact test compared the number of males versus females with an impaired DLCO defined as below the lower limit of normal (< LLN, < 5th percentile). A Fisher’s exact test also compared the number of males versus females with a restrictive spirometric pattern as defined by the ratio of the pre-bronchodilator forced expiratory volume in 1 s (FEV_1_) to forced vital capacity (FVC) being ≥ LLN with the FVC being less than the LLN [35]. The Fisher's Exact Test would allow an examination of sex differences in the proportion of males versus females with abnormally low lung function. Several N-1 Chi-Squared Tests were used to determine whether the percentage of several signs and symptoms present post-COVID-19 were different between those with a DLCO < LLN and those with a DLCO ≥ LLN [36]. A Benjamini–Hochberg procedure was used to control the false discovery rate [37], which we set to 0.05.

Binary logistic regression was performed using the backward: likelihood ratio method. This stepwise method enters all independent variables at once and then removes each variable one at a time according to the probability of the likelihood-ratio statistic until only the significant variables remain in the model. Binary logistic regression was used to determine the factors associated with an impaired DLCO (DLCO < LLN, less than a z-score of -1.645, or < 5th percentile) in patients with previous mild COVID-19. Cardiovascular disease (CVD) risk factors [1 = yes, 0 = no for smoking status, high blood pressure, cardiac arrhythmia, and obesity], true cardiac disease (yes = 1, no = 0), fatigue (yes = 1, no = 0), dyspnoea (yes = 1, no = 0), cough (yes = 1, no = 0), headache (yes = 1, no = 0), chest tightness (yes = 1, no = 0), sore throat (yes = 1, no = 0), persistent loss of smell (yes = 1, no = 0), dysfunction in the sense of taste (yes = 1, no = 0), conjunctivitis (yes = 1, no = 0), blocked and/or runny nose (yes = 1, no = 0), use of oral corticosteroids during the disease (yes = 1, no = 0), use of anticoagulant medications (yes = 1, no = 0), sex (male = 1, female = 0), age (years old), height (cm), weight (kg), body mass index (BMI, kg/m^2^), a restrictive spirometric pattern (forced vital capacity or FVC below the lower limit of normal, LLN, and FEV_1_/FVC ≥ LLN, yes = 1, no = 0), forced expiratory volume in 1 s (FEV_1_, L), forced expiratory flow rate over the middle half of expiration (FEF_25-75_, L/s) and peak expiratory flow (PEF, L/s).

Using mean data from 22 previous studies (including the current study), multiple linear regression analysis using forward selection was used to identify which of the five following factors would predict the proportion of patients who had previous COVID-19 and impaired DLCO at follow-up. The mean age (years old), mean body mass index (kg/m^2^), the mean number of days between receiving the COVID-19 diagnosis and follow-up, and history of mild vs severe COVID-19 disease (i.e. patients that were either hospitalised, intubated, presented with fibrotic C.T. changes in the lung were labelled as 1 or severe, compared to those that were not, and labelled as 0 or mild), and the criteria used to define impaired DLCO (DLCO < 80% predicted labelled as 1, vs DLCO < LLN labelled as 0) were predictors used in the model.

All data were analysed by a statistical software package (IBM SPSS Statistics, Version 27, Chicago IL). A *p*-value of < 0.05 was used to signify statistical significance. Any case with a standardised residual ≥ 3.0 was removed from any model.

## Results

One hundred and fifty subjects were recruited from the Long-term follow-up COVID-19 clinic. Four patients were removed due to the reference equations not fitting the age range of the subjects, leaving 146 patients for the analysis. The anthropometric characteristics are presented in Table 1. Approximately 50% of the subjects were obese (BMI ≥ 30 kg/m^2^). The gas exchange parameter, DLCO, was reduced compared to predicted values (Table 2). In fact, 30% of the sample had a DLCO value below the LLN, and the percentages were similar between males and females (Table 3). Twenty-one percent of the sample had a restrictive spirometric pattern (FVC < LLN and an FEV_1_/FVC ≥ LLN) (Table 3), and when coupled with a DLCO < LLN, about 13% of the patients had both impaired DLCO and lung restriction.

**Table 1:** Anthropometric and lung function data post mild COVID-19

	Males (n = 59)	Females (n = 87)
Age (yrs)	50 (13) [28–82]	50 (14) [25–83]
Weight (kg)	84.6 (15.0) [61.0–133.0]	70.0 (15.3) [42.0–118.0]
Height (cm)	168 (8) [150–182]	153 (8) [137–169]
Body mass index (kg/m^2^)	29.9 (4.0) [22.2–42.0]	30.0 (6.2) [16.6–44.1]
FVC (L)	3.97 (1.00) [1.20–6.73]	2.16 (0.66) [1.18–4.51]
FEV_1_ (L)	3.26 (0.81) [1.06–5.18]	2.14 (0.59) [0.88–3.75]
FEV_1_/FVC	0.82 (0.05) [0.64–0.91]	0.82 (0.06) [0.61–0.95]
PEF (L/s)	9.26 (2.15) [3.29–14.91]	5.75 (1.73) [2.07–9.70]
DLCO (mL min^−1^ mmHg^−1^)	26.6 (7.7) [7.6–53.6]	18.7 (4.8) [7.3–30.8]
VA (L)	6.03 (1.43) [1.88–9.53]	4.36 (0.89) [1.92–6.25
*K*CO mL min^−1^ mmHg^−1^ L^−1^	4.44 (0.91) [2.46–6.84]	4.31 (0.84) [1.71–7.04]
Altitude of testing (m)	7	7

Data are showed as mean (S.D.). Brackets represent ranges

**Table 2:** Percent predicted for several lung function indices

	Males (n = 59)	Females (n = 87)
% predicted FVC	91 (16) [31–127]	93 (14) [61–131]
% predicted FEV_1_	95 (17) [34–124]	90 (14) [56–120]
% predicted FEV_1_/FVC	104 (6) [83–116]	101 (6) [78–116]
% predicted PEF	107 (24) [49–158]	105 (28) [53–180]
% predicted DLCO	81 (19) [25–136]	78 (17) [39–153]

Data are shown as mean (S.D.). Brackets represent ranges

**Table 3:** The number and percentage of males and females below the lower limit of normal (< 5th percentile)

	Males (n = 59)	Females (n = 87)	Total (n = 146)
FVC	15 (25%)	18 (21%)	33 (23%)
FEV_1_	11 (19%)	14 (16%)	25 (17%)
FEV_1_/FVC	1 (2%)	3 (3%)	4 (3%)
PEF	6 (10%)	9 (10%)	15 (10%)
DLCO	16 (27%)	28 (32%)	44 (30%)

A Fisher's Exact test was used to compare frequencies between males and females. No statistically significant differences were noted between males and females

For the regression analysis, two subjects had missing data, and then data from an additional five subjects were removed from the analysis, with their data being outliers (standardised residuals ≥ 3.0). Thus 139 subjects remained for the binary logistic regression analysis. The analysis revealed an overall model of four predictors that were statistically reliable in distinguishing between those with a DLCO below the LLN and those with normal DLCO [-2 Log-Likelihood = 101.7, Nagelkerke R^2^ = 0.51; Omnibus tests of model coefficients χ^2^ = 60.0, d*f* = 4, *p* < 0.001, Akaike Information Criterion (AIC) = 111; Bayesian Information Criterion (BIC) = 126]. Increased age (from 25 to 83 years of age) and a restrictive spirometric pattern increased the probability of an impaired DLCO, while a blocked / runny nose and excessive sweating reduced the probability of having an impaired DLCO. The model was a good fit [Hosmer and Lemeshow Test, χ^2^ = 6.7, df = 8, *p* = 0.57], correctly identifying 81% of the cases (Table 4).

**Table 4:** Variables that predict impaired DLCO in mild COVID-19 patients (n = 139 patients)

							95% CI for odds ratio
Variables	B	S.E	Wald	d_f_	Sig	Odds ratio	lower–upper
Age	0.094 [0.053, 0.143]	0.02	17.0	1	0.000	1.10	1.05 to 1.15
Restriction (1 = yes, 0 = no)	2.49 [1.34, 3.79]	0.62	16.3	1	0.000	12.1	3.61 to 40.41
Blocked nose (yes = 1; no = 0)	− 2.29 [− 3.79, − 1.05]	0.69	11.0	1	0.001	0.10	0.03 to 0.39
Excessive sweating (yes = 1, no = 0)	− 2.51 [− 4.43, − 0.92]	0.89	8.0	1	0.005	0.08	0.01 to 0.46
Constant	− 5.91 [− 8.79, − 3.54]	1.33	19.8	1	0.000	0.003	0.0002 to 0.0365

Brackets represent the 95% CI. The model was accurate 81% of the time, i.e., correctly classifying 81% of the cases [85% (93/110) of the negative cases (true negative rate, specificity) and 69% (20/29) of the positive cases (true positive rate, sensitivity). The false-positive rate was 46%, and the false-negative rate was 9%. The positive predictive value was 54%, and the negative predictive value was 91%. The area under the curve was 0.89 (Standard error = 0.03) 95% CI = 0.83 to 0.94). Seven outliers (5%) were removed (Standardised residuals ≥ 3.0)

Using mean data from 21 previous studies (including the current study, see Additional file 1: Table S1), multiple linear regression analysis was used to identify which factors would predict the proportion of previously infected SARS-CoV-2 patients with impaired DLCO at follow-up. Regression results indicate an overall model of four predictors (previous history of severe COVID-19, criteria used to define impaired DLCO, mean age of the group, and number of days between diagnosis of COVID-19 and testing) that significantly determined the percentage of previously infected COVID-19 patients with an impaired DLCO at follow-up [R^2^_adj_ = 0.46, F(4,31) = 8.4, residual standard deviation = 15.6%, *p* < 0.001, AIC = 306, BIC = 316]. The model accounted for 46% of the variance defining those with DLCO impairment. A summary of the regression model is presented in Table 5. Having a previous severe case of COVID-19 increases the proportion of those with impaired DLCO by 21% at follow-up. When using the more liberal definition of impaired DLCO (< 80% of predicted), the proportion of those with impaired DLCO increased by 13% in a given study. Finally, when the patient's mean age for a study increased by one year (from 41 to 69 years), the proportion of those with impaired DLCO increased by about 1%. Interestingly, the time of follow-up (20 to 180 days) did not seem to affect the percentage of those with impaired DLCO in a particular study.

**Table 5:** Variables that predict the proportion of patients who had previous COVID-19 disease with an impaired DLCO at follow-up using the mean data from 21 previous studies

Model	Unstandardized coefficient B	Coefficient S.E	Standardized coefficient Beta	t	Sig	95% confidence interval for B
Constant	− 33.47	21.08		− 1.59	0.12	− 76.5 to 9.52
History of mild vs severe COVID-19	20.55	5.26	0.49	3.91	< 0.001	0.97 to 1.03
Mean age of study (years old)	1.21	0.38	0.40	3.14	0.004	0.95 to 1.05
Criteria to define impaired DLCO	13.13	5.23	0.32	2.51	0.017	0.99 to 1.01
Mean number of days between receiving a diagnosis of COVID-19 and testing	− 0.12	0.68	− 0.23	− 1.76	0.088	0.94 to 1.06

Where mild COVID-19 = 0; Severe COVID-19 = 1; impaired DLCO defined by DLCO < LLN = 0; DLCO defined by being less than 80% of predicted = 1

## Discussion

The main purpose of this study was to identify possible predictors of impaired DLCO in patients that had mild COVID-19 within the first 4 to 6 weeks. We found that patients who are recovering from mild COVID-19 also have persistent symptoms (Additional file 1: Table S2) as well as pulmonary function abnormalities (Table 3). For each one-year increase in age, the odds of having an impaired DLCO increased by 10%, while having a restrictive spirometric pattern increased the risk of having an impaired DLCO by 12-fold (Table 4). Regarding symptoms, having reported a blocked/runny nose and excessive sweating at follow-up reduced the odds of having an impaired DLCO by about 90% (Table 4). When using the logistic model, with a mean age of 50.7 years, having no lung restriction (0), no blocked nose (0), and no excessive night sweats (0), the probability of an impaired DLCO in this population is 24%. The chance increases to 79% when a restrictive spirometric pattern is evident (Table 4). Thus, having a restrictive spirometric pattern increases the odds of having impaired DLCO at follow-up by 12-fold. While 20% per cent of the patients had a restrictive spirometric pattern, only 3% of patients showed an obstructive pattern (as defined by an FEV_1_/FVC below LLN), demonstrating the after-effects COVID-19 are likely to result in lung restriction and poor pulmonary diffusion. A binary logistic regression model was chosen because it determined how well the measured variables predicted impaired DLCO while providing a summary of the accuracy of the classification of cases. The data collected had many variables, and the logistic regression analysis allowed the determination of the most important factors that predicted impaired DLCO in these patients.

The most common lung function parameter that is impaired in COVID-19 survivors is DLCO [38]. We have identified, in our population, 3 out of 10 patients without pneumonia or reduced SpO_2_ beyond 90% on room air at sea level (Yucatán, México) have an abnormal lung diffusion during the follow-up. Current available information is focused on severe cases and less attention has been paid to mild cases. Yet, these patients might have lung function abnormalities since mild cases usually develop ground glass opacities instead of lung consolidation. Ground glass opacities are associated with local dysregulations involving endothelial and epithelial injury markers suggesting some degree of venous thromboembolism, endothelial dysfunction, and abnormalities in cardiopulmonary circulatory physiology, which in turn may reflect DLCO abnormalities [39]. In a recent meta-analysis of 12 studies, being female, altered chest computerised tomography, age, higher D-dimer levels, and urea nitrogen were identified as factors for impaired DLCO [38]. This current study demonstrated similar odds ratios for age as the meta-analysis [38]; however, unlike the meta-analysis, sex was not a predictor of impaired DLCO in this study. Furthermore, the meta-analysis did not report that blocked / runny noses or excessive sweating were negative predictors. The results demonstrating that excessive sweating and runny noses were protective against impaired gas exchange are unique and puzzling and could be spurious outcomes. From the data presented in Additional file 1: Table S2, the proportion of those with a runny nose and abnormal sweating was statistically significant between those with impaired DLCO compared to those with normal DLCO. However, when the Benjamini–Hochberg procedure was used to control the false-discovery rate for 25 paired comparisons, these two variables became non-significant.

Another purpose of this study was to determine the variables that predict the percentage of previously infected SARS-CoV-2 patients who had a DLCO impairment during follow-up. Patients with previous severe COVID-19 disease at diagnosis would increase the likelihood of impaired DLCO by nearly 21% compared to those with previous mild COVID-19 disease. When studies used the usual cut-off < 80% of predicted to define DLCO impairment, then 13% more patients would be classified as having an abnormal gas exchange compared to if DLCO impairment was defined as below the LLN. Thus, if using the stricter definition of DLCO impairment as being below the LLN (i.e., below the 5th percentile) for height, age, sex, and ethnicity, 13% fewer patients would be classified as having a reduced DLCO. The definition of a low DLCO being < 80% of predicted is not correct and may misclassify patients, as the per cent of the predicted value at the LLN (5th percentile) decreases beginning at about 40 years of age [40, 41]. Patients with the same height, sex, and ethnicity have a DLCO of about 79% predicted at 40 years of age at the LLN (5th percentile) compared to about 73% predicted at 85 years of age at the LLN [41]. Therefore, using z-scores is preferred instead of using an absolute cut-off of less than 80% predicted to define a clinically low DLCO.

We were not able to measure total lung capacity (TLC) using a body plethysmograph to verify lung restriction; thus, a restrictive spirometric pattern (FEV_1_/FVC ≥ LLN and FVC < LLN, pre-bronchodilator) was used instead as a surrogate of true lung restriction. The sensitivity to identify true pulmonary restriction (TLC < LLN) with a restrictive spirometric pattern is about 34%, but the specificity is nearly 98% [42]. The negative predictive value (NPV) means that the percentage of patients who do not have a restrictive spirometric pattern and do not have restrictive lung disease is 97% [39]. The prevalence of a restrictive spirometric pattern (FEV_1_/FVC ≥ LLN and FVC < LLN, pre-bronchodilator) in populations is about 3 to 9% [35, 42]. In this group of patients with mild COVID-19, we found the restrictive spirometric pattern to be about 20% which is more than double the population average.

Among persistent symptoms, fatigue and shortness of breath on effort are the most prevalent descriptors included in Long COVID-19, and these were not different in ambulatory patients recovering from mild COVID-19 [30]. About 74% of the patients experienced undue fatigue, and nearly half experienced shortness of breath on effort and/or a significant cough.

## Limitations

Our study has some limitations. In addition to not having measured lung volumes with plethysmography, we do not have any baseline lung function data in these patients prior to SARS-CoV-2 infection or a control group due to the pandemic to perform further analysis. However, most studies involving lung function analysis among COVID-19 survivors were done with the same limitation. Indeed, it is difficult to determine with certainty that these patients with poor diffusing capacity and/or a restrictive spirometric pattern are due to them having mild COVID-19. Nonetheless, these patients are compared against established reference norms. The prevalence of an impaired DLCO in our sample was five times more than expected in a normal population and at least two-fold more than expected for a restrictive spirometric pattern. To reduce this bias, we analysed all risk factors related to lung function abnormalities, including published studies. Also, there is a gap in implementing regular lung function assessments in suspected obstructive lung disease cases. Instead, chest x-ray studies are more frequently requested, especially in countries with limited-resource settings; therefore, it is expected that patients were not subjected to pulmonary function tests prior to SARS-CoV-2 infection.

## Conclusions

Nearly one-third of patients with mild COVID-19 have impaired DLCO 34 days post-diagnosis, and one-fifth of patients have lung restriction. The odds of having an impaired DLCO at follow-up increased by 10% for every one-year increase in age (from 25 to 83) and increased 12-fold if a restrictive spirometric pattern was evident. However, having excessive night sweats and a blocked/runny nose each reduced the probability of an impaired DLCO at follow-up by about 90%, demonstrating a protective effect against an impaired gas exchange. In a summary of 22 studies, having severe COVID-19 disease at diagnosis increased the percentage of those with impaired DLCO by 21%. And, if the study used < 80% of predicted to define DLCO impairment, then 13% more patients would be classified as having a poor gas exchange.

## Supplementary Information

**Additional file 1. Table S1.** Studies demonstrate impaired DLCO one to 180 days post- COVID-19 diagnosis. **Table S2.** Results from the medical assessment and detailed medical history obtained 34 (SD 4) days post-COVID-19 diagnosis.

## Data Availability

All data generated or analysed during this study are included in this article and/or its supplementary material files. The datasets used and/or analysed during the current study are available from the corresponding author on reasonable request.

## Abbreviations

SARS-CoV2: Severe acute respiratory syndrome coronavirus 2
COVID-19: Coronavirus disease 2019
DLCO: Pulmonary diffusing capacity for carbon monoxide
LLN: Lower limit of normality
WHO: World Health Organization
BMI: Body mass index
FEV_1_: Forced expiratory volume in 1 s
FVC: Forced vital capacity
FEF_25-75_: Forced expiratory flow rate over the middle half of expiration
PEF: Peak expiratory flow
TLC: Total lung capacity
CVD: Cardiovascular disease
