# Supplementary material for: Clinical predictors of lung function in patients recovering from mild COVID-19
Source: BMC Pulm Med. 2022 Jul 31;22:294. doi: 10.1186/s12890-022-02086-9 (PMC9339191; doi:10.1186/s12890-022-02086-9)
Supplement: Supplementary file 1 — Additional file 1. Table S1. Studies demonstrate impaired DLCO one to 180 days post- COVID-19 diagnosis. Table S2. Results from the medical assessment and detailed medical history obtained 34 (SD 4) days post-COVID-19 diagnosis. [file 12890_2022_2086_MOESM1_ESM.docx]

**ADDITIONAL FILE 1**

**Clinical predictors of lung function in patients recovering from mild COVID-19**

Arturo Cortes-Telles^1*^ ORCID: **0000-0002-5322-5604**

Esperanza Figueroa-Hurtado^1^ ORCID: **0000-0003-1388-0572**

Diana Lizbeth Ortiz-Farias^1^ ORCID: **0000-0002-3212-1929**

Gerald Stanley Zavorsky^2*^ ORCID: **0000-0002-4473-1601**

^1^ Respiratory and Thoracic Surgery Unit. Hospital Regional de Alta Especialidad de la Peninsula de Yucatan. Yucatan, Mexico.

^2^Department of Physiology and Membrane Biology, University of California, Davis, United States of America.

^*^Both share first authorship.

This supplement is peer-reviewed.

**Table S1.** Studies demonstrate impaired DLCO one to 180 days post- COVID-19 diagnosis.

| Study | # Of patients | Age (years) | BMI | Number of days post-COVID-19 | Main findings |
| --- | --- | --- | --- | --- | --- |
| DLCO below the LLN |  |  |  |  |  |
| Present Study | 146 | 50 (14) | 30 (5) | 34 (4) days | 30% with mild COVID-19 presented with DLCO values below the LLN |
| Abdallah *et al.* (2021)[1] | 63 | 49 (13) | 29 (8) | 120 (16) days | 71% of patients hospitalized for COVID-19 had a DLCO < LLN. However, 32% of the non-hospitalized COVID-19 patients had a DLCO < LLN |
| Van der Sar et al. (2021)[2] | 101 | 66 (13) | 27 | 42 days | 63% of patients hospitalized with severe pneumonia has a DLCO < LLN. However, 52% of those with moderate pneumonia had a DLCO < LLN |
| Morin *et al.* (2021) [3] | 152 | 61 (16) | 29 (6) | 120 days | 22% in total were below the LLN for DLCO. 16% of non-intubated patients were below the LLN, vs 36% of patients intubated |
| Cortés-Telles *et al.* (2021)[4] | 186 | 47 (13) | 32 (7) | 59 (13) | 19% with mild/moderate COVID-19 illness were below the LLN for DLCO, compared to 54% for those with severe/critical COVID-19 illness. |
| Goachicoa-Rangel *et al.* (2021)[5] | 171 | 46 (12) | 29 (5) | 84 (20) days | 17% of those without invasive ventilation and 41% with invasive ventilation presented with DLCO values below the LLN |
| Nunez-Fernandez *et al.* (2021)[6] | 200 | 62 | 29 | 120 days | 17% presented with DLCO values below the LLN |
| Ekbom *et al.* (2021)[7] | 60 | N/A | ⁓30 | 120 days | 45% presented with DLCO values below the LLN |
| Ven den Borst *et al.* (2021)[8] | 124 | 59 (14) | 28 (5) | 90 days | 55%, 33%, and 0% of patients with severe, moderate, and mild disease, respectively were below the LLN for DLCO |
| Barisione & Brusasco (2021)[9] | 94 | 61 (12) | 29 (4) | 90 days | 20% presented with DLCO values below the LLN |
| Lerum *et al.* (2020)[10] | 102 | 54 (5) | 26 | 90 days | 24% presented with DLCO values below the LLN. 29% were admitted to ICU. 23% were not admitted. |
| DLCO < 80% predicted |  |  |  |  |  |
| Guler *et al.* (2021) [11] | 113 | 57 (11) | 28 (11) | 128 days | 13% and 83% of those with mild and severe COVID-19 illness had a DLCO < 80% of predicted. |
| Sonnweber *et al.* (2021)[12] | 145 | 57 (14) | 26 (5) | 63 (23)  103 (21) | 31% and 21% were presented with a DLCO < 80% predicted at 63 days and 103 days post-COVID-19, respectively. |
| Han *et al.* (2021)[13] | 114 | 54 (12) | -- | 180 days | 26% of all patients presented with a DLCO of < 80% predicted. 50% of patients with fibrotic-like changes on CT presented with a DLCO < 80% of predicted vs 13% of patients without fibrotic-like changes on CT |
| Bellan *et al.* (2021)[14] | 219 | 61 | -- | 120 days | 52% of patients with COVID-19 presented with DLCO values < 80% predicted |
| Qin *et al.* (2021)[15] | 81 | 59 (14) | 24 (3) | 90 days | 42% and 68% of those with non-severe and severe COVID-19, respectively had a DLCO < 80% pred. |
| Blanco *et al.* (2021)[16] | 108 | 55 (11) | -- | 104 | 52% with DLCO values below 80% predicted |
| Shah et al. (2020) [17] | 60 | 67 | 25 | 82 days | About 50% of subjects were below 80% predicted. |
| Mo *et al.* (2020) [18] | 110 | 49 (14) | 24 (3) | 20 (6) days  34 (7) days | 30% and 84% of those with mild and severe COVID-19 illness had a DLCO < 80% of predicted. |
| Liang *et al.* (2020) [19] | 76 | 41 (14) | 24 (5) | 30 days | 20% of patients presented with a DLCO of < 80% predicted |
| Huang et al. (2020)[20] | 57 | 47 (14) | 24 (4) | 30 days | 77% and 43% of those with severe and non-severe COVID-19 illness had a DLCO < 80% of predicted. |
| Liu et al. (2020)[21] | 36 | 69 (8) | 23 (4) | unsure | About 85% had DLCO values below 80% predicted |
| Zhao *et al.* (2020)[22] | 55 | 48 (16) | 25 (3) | 90 days | 24% presented with DLCO values below the LLN |

**Table S2.** Results from the medical assessment and detailed medical history obtained 34 (SD 4) days post-COVID-19 diagnosis.

|  | DLCO < LLN  N = 44 | DLCO ≥ LLN  N = 100 | p-value |
| --- | --- | --- | --- |
| Restrictive spirometric pattern (FVC< LLN & FEV_1_/FVC ≥ LLN) | 18 (41%) | 13 (13%) | **0.0002** |
| Weight loss | 16 (36%) | 40 (40%) | 0.65 |
| Use of oral corticosteroids | 9 (20%) | 14 (14%) | 0.37 |
| Use of anticoagulants | 34 (77%) | 75 (75%) | 0.80 |
| Cardiac disease | 3 (7%) | 1 (1%) | 0.046 |
| Tachycardia | 1 (2%) | 6 (6%) | 0.30 |
| Symptoms of Cardiovascular Disease |  |  |  |
| Hypertension | 11 (25%) | 17 (17%) | 0.27 |
| Diabetes | 1 (2%) | 4 (4%) | 0.54 |
| Current or former smoker | 7 (16%) | 14 (14%) | 0.76 |
| Obesity (BMI ≥ 30 kg/m^2^) | 25 (57%) | 44 (44%) | 0.15 |
| Other symptoms post-COVID-19 diagnosis |  |  |  |
| Fatigue | 34 (77%) | 73 (73%) | 0.61 |
| Shortness of breath on effort | 23 (52%) | 38 (38%) | 0.12 |
| Chest tightness | 15 (34%) | 37 (37%) | 0.73 |
| Sore throat | 13 (30%) | 26 (26%) | 0.62 |
| Headache | 6 (14%) | 14 (14%) | 1.00 |
| Loss of smell | 6 (14%) | 22 (22%) | 0.27 |
| Loss of taste | 4 (9%) | 18 (18%) | 0.17 |
| Abdominal pain | 1 (2%) | 5 (5%) | 0.40 |
| Muscle or joint pain | 6 (14%) | 18 (18%) | 0.56 |
| Visual signs present post-COVID-19 diagnosis |  |  |  |
| Cough | 20 (46%) | 43 (43%) | 0.74 |
| Hair loss | 11 (25%) | 28 (28%) | 0.71 |
| Diarrhea | 4 (9%) | 6 (6%) | 0.52 |
| Blocked nose and/or runny nose | 9 (21%) | 44 (44%) | 0.0087 |
| Abnormal sweating | 5 (11%) | 25 (25%) | 0.057 |
| Sore or red eyes | 4 (9%) | 4 (4%) | 0.23 |

Patients were asked to recount presence or absence of symptoms at time of visit. The N-1 Chi-Squared Test was used to compare proportions between those with a DLCO < LLN and those with a DLCO ≥ LLN [23]. A Benjamini-Hochberg procedure was used to control the false discovery rate [24], which we set to 0.05. As such, the only comparison that were significantly different between the two groups was the proportion of those with a restrictive spirometric pattern.

**References**

1. Abdallah SJ, Voduc N, Corrales-Medina VF, McGuinty M, Pratt A, Chopra A, Law A, Garuba HA, Thavorn K, Reid RER, Lavoie KL, Crawley A, Chirinos JA, Cowan J. Symptoms, Pulmonary Function and Functional Capacity Four Months after COVID-19. *Ann Am Thorac Soc* 2021.

2. van der Sar-van der Brugge S, Talman S, Boonman-de Winter L, de Mol M, Hoefman E, van Etten RW, De Backer IC. Pulmonary function and health-related quality of life after COVID-19 pneumonia. *Respir Med* 2021: 176: 106272.

3. Morin L, Savale L, Pham T, Colle R, Figueiredo S, Harrois A, Gasnier M, Lecoq AL, Meyrignac O, Noel N, Baudry E, Bellin MF, Beurnier A, Choucha W, Corruble E, Dortet L, Hardy-Leger I, Radiguer F, Sportouch S, Verny C, Wyplosz B, Zaidan M, Becquemont L, Montani D, Monnet X. Writing Committee for the Comebac Study Group. Four-Month Clinical Status of a Cohort of Patients After Hospitalization for COVID-19. *JAMA* 2021: 325(15): 1525-1534.

4. Cortes-Telles A, Lopez-Romero S, Figueroa-Hurtado E, Pou-Aguilar YN, Wong AW, Milne KM, Ryerson CJ, Guenette JA. Pulmonary function and functional capacity in COVID-19 survivors with persistent dyspnoea. *Respir Physiol Neurobiol* 2021: 288: 103644.

5. Gochicoa-Rangel L, Hernandez-Morales AP, Salles-Rojas A, Madrid-Mejia W, Guzman-Valderrabano C, Gonzalez-Molina A, Salas-Escamilla I, Duran-Cuellar A, Silva-Ceron M, Hernandez-Morales V, Reyes-Garcia A, Alvarado-Amador I, Lozano-Martinez L, Enright P, Pensado-Piedra LE, Torre-Bouscoulet L. Gas Exchange Impairment During COVID-19 Recovery. *Respir Care* 2021: 66(10): 1610-1617.

6. Nunez-Fernandez M, Ramos-Hernandez C, Garcia-Rio F, Torres-Duran M, Nodar-Germinas A, Tilve-Gomez A, Rodriguez-Fernandez P, Valverde-Perez D, Ruano-Ravina A, Fernandez-Villar A. Alterations in Respiratory Function Test Three Months after Hospitalisation for COVID-19 Pneumonia: Value of Determining Nitric Oxide Diffusion. *J Clin Med* 2021: 10(10).

7. Ekbom E, Frithiof R, Emilsson O, Larson LM, Lipcsey M, Rubertsson S, Wallin E, Janson C, Hultstrom M, Malinovschi A. Impaired diffusing capacity for carbon monoxide is common in critically ill Covid-19 patients at four months post-discharge. *Respir Med* 2021: 182: 106394.

8. van den Borst B, Peters JB, Brink M, Schoon Y, Bleeker-Rovers CP, Schers H, van Hees HWH, van Helvoort H, van den Boogaard M, van der Hoeven H, Reijers MH, Prokop M, Vercoulen J, van den Heuvel M. Comprehensive Health Assessment 3 Months After Recovery From Acute Coronavirus Disease 2019 (COVID-19). *Clin Infect Dis* 2021: 73(5): e1089-e1098.

9. Barisione G, Brusasco V. Lung diffusing capacity for nitric oxide and carbon monoxide following mild-to-severe COVID-19. *Physiol Rep* 2021: 9(4): e14748.

10. Lerum TV, Aalokken TM, Bronstad E, Aarli B, Ikdahl E, Lund KMA, Durheim MT, Rodriguez JR, Meltzer C, Tonby K, Stavem K, Skjonsberg OH, Ashraf H, Einvik G. Dyspnoea, lung function and CT findings 3 months after hospital admission for COVID-19. *Eur Respir J* 2021: 57(4).

11. Guler SA, Ebner L, Aubry-Beigelman C, Bridevaux PO, Brutsche M, Clarenbach C, Garzoni C, Geiser TK, Lenoir A, Mancinetti M, Naccini B, Ott SR, Piquilloud L, Prella M, Que YA, Soccal PM, von Garnier C, Funke-Chambour M. Pulmonary function and radiological features 4 months after COVID-19: first results from the national prospective observational Swiss COVID-19 lung study. *Eur Respir J* 2021: 57(4).

12. Sonnweber T, Sahanic S, Pizzini A, Luger A, Schwabl C, Sonnweber B, Kurz K, Koppelstatter S, Haschka D, Petzer V, Boehm A, Aichner M, Tymoszuk P, Lener D, Theurl M, Lorsbach-Kohler A, Tancevski A, Schapfl A, Schaber M, Hilbe R, Nairz M, Puchner B, Huttenberger D, Tschurtschenthaler C, Asshoff M, Peer A, Hartig F, Bellmann R, Joannidis M, Gollmann-Tepekoylu C, Holfeld J, Feuchtner G, Egger A, Hoermann G, Schroll A, Fritsche G, Wildner S, Bellmann-Weiler R, Kirchmair R, Helbok R, Prosch H, Rieder D, Trajanoski Z, Kronenberg F, Woll E, Weiss G, Widmann G, Loffler-Ragg J, Tancevski I. Cardiopulmonary recovery after COVID-19: an observational prospective multicentre trial. *Eur Respir J* 2021: 57(4).

13. Han X, Fan Y, Alwalid O, Li N, Jia X, Yuan M, Li Y, Cao Y, Gu J, Wu H, Shi H. Six-month Follow-up Chest CT Findings after Severe COVID-19 Pneumonia. *Radiology* 2021: 299(1): E177-E186.

14. Bellan M, Soddu D, Balbo PE, Baricich A, Zeppegno P, Avanzi GC, Baldon G, Bartolomei G, Battaglia M, Battistini S, Binda V, Borg M, Cantaluppi V, Castello LM, Clivati E, Cisari C, Costanzo M, Croce A, Cuneo D, De Benedittis C, De Vecchi S, Feggi A, Gai M, Gambaro E, Gattoni E, Gramaglia C, Grisafi L, Guerriero C, Hayden E, Jona A, Invernizzi M, Lorenzini L, Loreti L, Martelli M, Marzullo P, Matino E, Panero A, Parachini E, Patrucco F, Patti G, Pirovano A, Prosperini P, Quaglino R, Rigamonti C, Sainaghi PP, Vecchi C, Zecca E, Pirisi M. Respiratory and Psychophysical Sequelae Among Patients With COVID-19 Four Months After Hospital Discharge. *JAMA Netw Open* 2021: 4(1): e2036142.

15. Qin W, Chen S, Zhang Y, Dong F, Zhang Z, Hu B, Zhu Z, Li F, Wang X, Wang Y, Zhen K, Wang J, Wan Y, Li H, Elalamy I, Li C, Zhai Z, Wang C. Diffusion capacity abnormalities for carbon monoxide in patients with COVID-19 at 3-month follow-up. *Eur Respir J* 2021: 58(1).

16. Blanco JR, Cobos-Ceballos MJ, Navarro F, Sanjoaquin I, Arnaiz de Las Revillas F, Bernal E, Buzon-Martin L, Viribay M, Romero L, Espejo-Perez S, Valencia B, Ibanez D, Ferrer-Pargada D, Malia D, Gutierrez-Herrero FG, Olalla J, Jurado-Gamez B, Ugedo J. Pulmonary long-term consequences of COVID-19 infections after hospital discharge. *Clin Microbiol Infect* 2021: 27(6): 892-896.

17. Shah AS, Wong AW, Hague CJ, Murphy DT, Johnston JC, Ryerson CJ, Carlsten C. A prospective study of 12-week respiratory outcomes in COVID-19-related hospitalisations. *Thorax* 2021: 76(4): 402-404.

18. Mo X, Jian W, Su Z, Chen M, Peng H, Peng P, Lei C, Chen R, Zhong N, Li S. Abnormal pulmonary function in COVID-19 patients at time of hospital discharge. *Eur Respir J* 2020: 55(6).

19. Liang L, Yang B, Jiang N, Fu W, He X, Zhou Y, Ma WL, Wang X. Three-month Follow-up Study of Survivors of Coronavirus Disease 2019 after Discharge. *J Korean Med Sci* 2020: 35(47): e418.

20. Huang Y, Tan C, Wu J, Chen M, Wang Z, Luo L, Zhou X, Liu X, Huang X, Yuan S, Chen C, Gao F, Huang J, Shan H, Liu J. Impact of coronavirus disease 2019 on pulmonary function in early convalescence phase. *Respir Res* 2020: 21(1): 163.

21. Liu K, Zhang W, Yang Y, Zhang J, Li Y, Chen Y. Respiratory rehabilitation in elderly patients with COVID-19: A randomized controlled study. *Complement Ther Clin Pract* 2020: 39: 101166.

22. Zhao YM, Shang YM, Song WB, Li QQ, Xie H, Xu QF, Jia JL, Li LM, Mao HL, Zhou XM, Luo H, Gao YF, Xu AG. Follow-up study of the pulmonary function and related physiological characteristics of COVID-19 survivors three months after recovery. *EClinicalMedicine* 2020: 25: 100463.

23. Campbell I. Chi-squared and Fisher-Irwin tests of two-by-two tables with small sample recommendations. *Stat Med* 2007: 26(19): 3661-3675.

24. Benjamini Y, Hochberg Y. Controlling the false discovery rate: a practical and powerful approach to multiple testing. *J Royal Stat Soc B* 1995: 57(1): 289-300.
